# Supplementary material for: End Sequence Analysis Toolkit (ESAT) expands the extractable information from single-cell RNA-seq data
Source: Genome Res. 2016 Oct;26(10):1397–410. doi: 10.1101/gr.207902.116 (PMC5052061; doi:10.1101/gr.207902.116)
Supplement: Supplemental Material [file supp_gr.207902.116_Supp_Materials.tar.gz › ESAT-esat_v0.1/doc/html/classbroad_1_1core_1_1motif_1_1meme_1_1_meme_motif.html]

ESAT: broad.core.motif.meme.MemeMotif Class Reference


ESAT


- Main Page
- Related Pages
- Packages
- Classes
- Files

- Class List
- Class Index
- Class Hierarchy
- Class Members

All Classes Namespaces Files Functions Variables Enumerator Pages

- broad
- core
- motif
- meme
- MemeMotif

Classes |
Public Member Functions |
List of all members

broad.core.motif.meme.MemeMotif Class Reference

Collaboration diagram for broad.core.motif.meme.MemeMotif:

[legend]

|  |  |
| --- | --- |
| Classes | |
| class | **MatchedSequence** |
|  | |

|  |  |
| --- | --- |
| Public Member Functions | |
|  | MemeMotif (String string) |
|  | |
| String | getName () |
|  | |
| List< MatchedSequence > | getMatches () |
|  | |
| int | getWidth () |
|  | |
| int | getSites () |
|  | |
| float | getLlr () |
|  | |
| double | getEval () |
|  | |
| void | addMatch (Sequence match, int startInScannedSequence, double pval) |
|  | |
| void | addPWMColumn (double[] col) |
|  | |
| PositionWeightMatrix | getPWM () |
|  | |

## Constructor & Destructor Documentation

|  |  |  |  |  |  |
| --- | --- | --- | --- | --- | --- |
| broad.core.motif.meme.MemeMotif.MemeMotif | ( | String | *string* | ) |  |

## Member Function Documentation

|  |  |  |  |
| --- | --- | --- | --- |
| void broad.core.motif.meme.MemeMotif.addMatch | ( | Sequence | *match*, |
|  |  | int | *startInScannedSequence*, |
|  |  | double | *pval* |
|  | ) |  |  |

|  |  |  |  |  |  |
| --- | --- | --- | --- | --- | --- |
| void broad.core.motif.meme.MemeMotif.addPWMColumn | ( | double[] | *col* | ) |  |

|  |  |  |  |  |
| --- | --- | --- | --- | --- |
| double broad.core.motif.meme.MemeMotif.getEval | ( |  | ) |  |

|  |  |  |  |  |
| --- | --- | --- | --- | --- |
| float broad.core.motif.meme.MemeMotif.getLlr | ( |  | ) |  |

|  |  |  |  |  |
| --- | --- | --- | --- | --- |
| List<MatchedSequence> broad.core.motif.meme.MemeMotif.getMatches | ( |  | ) |  |

|  |  |  |  |  |
| --- | --- | --- | --- | --- |
| String broad.core.motif.meme.MemeMotif.getName | ( |  | ) |  |

|  |  |  |  |  |
| --- | --- | --- | --- | --- |
| PositionWeightMatrix broad.core.motif.meme.MemeMotif.getPWM | ( |  | ) |  |

|  |  |  |  |  |
| --- | --- | --- | --- | --- |
| int broad.core.motif.meme.MemeMotif.getSites | ( |  | ) |  |

|  |  |  |  |  |
| --- | --- | --- | --- | --- |
| int broad.core.motif.meme.MemeMotif.getWidth | ( |  | ) |  |

---

The documentation for this class was generated from the following file:

- java/broad/core/motif/meme/MemeMotif.java


---

Generated on Fri Aug 15 2014 15:10:54 for ESAT by  

 1.8.7
